# Living with type 1 diabetes as experienced by young adults

**DOI:** 10.1002/nop2.222

**Published:** 2018-12-18

**Authors:** Åsa Carlsund, Siv Söderberg

**Affiliations:** ^1^ Department of Nursing Sciences Mid‐Sweden University Östersund Sweden

**Keywords:** experience, nursing, qualitative study, type 1 diabetes, young adults

## Abstract

**Aim:**

Describe young adults` (19–30 years) experiences of living with Type 1 Diabetes (T1D).

**Background:**

Young adulthood is characterized by adaption to adult roles, gradual separation from parental support and leaving the parental home. Living with T1D in young adulthood raises challenges and concerns.

**Design:**

This study has a qualitative design.

**Methods:**

Semistructured interviews with 12 young adults living with T1D for 3–14 years, analysed with qualitative content analysis.

**Results:**

The analysis revealed contradictory ways of handling the illness, as is illuminated in two main categories (a) and five subcategories (b). Handling the situation and dealing with different opinions (a), (b) managing daily life, emotional roller coaster and general attitudes, own views and apprehensions, ignorance and lack of motivation. Most participants were motivated, had knowledge and were in control of their long‐term illness. Planning and structure were an essential part of their daily life. The participants were anxious about losing control of their bodies, the situation, die or be a burden to other people.

## 1 INTRODUCTION

Today Diabetes is one of the largest global health emergencies and among the top ten causes of death. Worldwide approximately 451 million people (aged 18–99 years) are estimated to have diabetes (International Diabetes Federation, 2017). In Sweden, there were approximately 43 thousand adult patients with T1D registered in the year of 2016, of them 9,831 were young adults (Swedish National Diabetes Register, 2016). In the past few decades, the annual incidence appears to be rising steadily by about 3% in high‐income countries (Dabelea, 2009). In addition to human effort and suffering, diabetes is tremendously expensive world wide. In 2017, the total healthcare expenditure was estimated to 958 billion US dollar, for people aged 18–99 years (International Diabetes Federation, 2017).

Type 1 Diabetes results from autoimmune destruction of insulin‐producing beta cells of the pancreas; therefore, insulin must be supplied in an alternative way (Daneman, 2006; WHO, 2016a, 2016b). One of the most important goals of T1D treatment is to strive for blood glucose levels as normal as possible (ADA, 2018; Swedish National Diabetes Register, 2016; WHO, 2016a). Blood glucose levels may be strongly affected by stress, anxiety and insulin treatment, for example, maintaining glycemic control at work or in school, hypoglycemia complications or keep track of needed supplies such as glucose‐testing materials or insulin (Cooper, Tekiteki, Khanolkar, & Braatvedt, 2016; Olshansky et al., 2008). In other words, self‐care is central for persons living with T1D (Helme & Grant Harrington, 2004; Hörnsten, Lundman, Selstam, & Sandström, 2005; Markowitz, Pratt, Aggarwal, Volkening, & Laffel, 2012; Martyn‐Nemeth, Schwarz Farabi, Mihailescu, Nemeth, & Quinn, 2016).

It is previously shown that living with a long‐term illness might lead to lack of energy, loneliness, isolation and depression (Fisher et al., 2015; Johnson et. al., 2012). Longitudinal studies (Bryden, Dunger, Mayou, Peveler, & Neil, 2003; Vanstone, Rewegan, Brundisini, Dejean, & Giacomini, 2015) imply that up to 50 per cent of young adults with T1D develop diabetes‐related complications, these complications include retinopathy, neuropathy and hypertension. Studies (Balfe, Brugha, et al., 2013; Hanna, 2014; Hirjaba, Häggman‐Laitila, Peitilä, & Kangasniemi, 2015) show that young adults with T1D have an increased mortality risk compared with the population in general. Further, people diagnosed in early childhood, or those with a history of significant hypoglycemia or hyperglycaemia show increased risk for difficulties with working memory and attention (Lin, Northam, Rankins, Werther, & Cameron, 2010).

The period between adolescence and young adulthood is fluent (Findley, Cha, Wong, & Faulkner, 2015). There are numerous developmental theories describing different periods of life, for example, adolescence, adulthood or senescence. According to a modern developmental theory, the period between 18 and 30 years defines the period of becoming an adult (Arnett, 2007). Earlier studies (Balfe, Brugha, et al., 2013; Berg‐Kelly, 2010; Berg‐ Kelly, 2011) entitles the biological age of 18 does not automatically mean that the person is able to take full responsibility for their health and wellbeing, particularly when living with a long‐term illness. Young adulthood is commonly characterized by adaption to adult roles, gradual separation from parental support and in most cases leaving the parental home (Lindberg & Söderberg, 2016; Peters & Laffel, 2011). In addition, living with a long‐term illness such as T1D might result in even more challenges (Balfe, Brugha, et al., 2013; Balfe, Doyle, et al., 2013; Garvey & Wolpert, 2011), that is, while young adults with T1D struggle to grow into adults’ roles they also struggle to take the entire responsibility of their diabetes management (Abdoli, Doosti Irani, Hardy, & Funnell, 2018; Abdoli, Hardy, & Hall, 2017).

Thus, the present study intends to increase the current knowledge of how young adults with T1D experience daily life. The knowledge gained by means of this study hopefully will strengthen opportunities to support and empower young adults with T1D to manage daily life. The aim of the study was to describe young adults’ experiences of living with T1D.

## 2 METHODS

### 2.1 Design

To enhance understanding of living with T1D a qualitative design was used. Semistructured interviews were conducted with the participants. To gain in‐depth information of living with T1D, the interview questions were constructed to cover different aspects of daily living (Polit & Beck, 2016).

### 2.2 Participants and Procedure

Twelve young adults (eight women and four men) with T1D were recruited with a purposive sampling method. The participants were recruited from a hospital in the middle of Sweden.

The criterion for participation was a diagnosis of T1D, age between 18 and 30 years, speak and understand the Swedish language. A diabetes nurse at the hospital mediated contact with patients that fulfilled the inclusion criterion. The nurse sent 70 invitation letters to presumptive participants and 12 returned the franked answering letters (Malterud, Siersma, & Guassora, 2016). To establish contact and decide time and place for the interview the presumptuous participants were contacted via telephone by the first author. In accordance with the participant's wishes, the interviews took place in a quiet room at the researchers’ workplace.

The participants were aged between 19–30 years (mean = 23) and had lived with the illness between 3–14 years (mean = 14). When they got the diagnosis of T1D, the participants were aged between 3–14 years (mean = 9.5). Eight lived in a joint household and four were single. Likewise, eight participants had a high school educational level and four had a university education.

### 2.3 Interviews

Individual, semistructured interviews (cf. Kvale & Brinkmann, 2009) were performed with the participants. The interviews lasted between 40–70 min (mean = 44). The young adults with T1D were asked to describe their experiences of “getting sick, living with the longcterm illness and being received and met by others”. Clarifying questions were asked when needed, for example, “What happened next?” “Can you give an example?” “How did you feel then?” The interviews were tape‐recorded and later transcribed verbatim. The interviews were numbered from 1 to 12 to place quotations in the results section.

### 2.4 Data analysis

To deepen the understanding of the young adults with T1D unique and still fragmented experience, the interviews were analysed according to the hands‐on qualitative content analysis guide as outlined by Elo and Kyngäs (2008). The first step was to organize the data. This process included open coding, that is, to describe as many aspects of the content as possible. Notes and headings were written in the text while reading it several times (Table 1). The codes were compared and contrasted, and the ones with similar meaning were put in the same subcategory. At the end of this stage, the researcher achieved a general description of the phenomenon. The names on the subcategories were put according to its lexical content. After that subcategories with similar content were grouped into main categories (Table 2). The abstraction process was pursued to the extent that was rational and feasible (Elo & Kyngäs, 2008).

**Table 1:** Data analysis process

Recommended stages by Elo and Kyngäs (2008)	Procedures done in present study
Open coding	Notes and headings were written in the text while reading it several times
Creating categories	The headings were transferred from the margins into coding sheets, ordered into subcategories aiming to collapse data
Abstracting	Subcategories with similar content were grouped into two main categories

**Table 2:** Overview of the main categories and subcategories

Main categories	Subcategories
Handling the situation	Managing daily life
	Emotional roller coaster
Dealing with different opinions	General attitudes
	Own views and apprehensions
	Ignorance and lack of motivation

### 2.5 Ethical considerations

Participants were informed about the nature of the study and were guaranteed confidentiality and anonymous presentation of the results. They were informed that their participation was voluntary, that they could withdraw from the study at any time. Written informed consent was obtained from all participants, to maintain anonymity and confidentiality during the entire process each consent was marked with a unique digit for each participant and the unique digit were kept during all stages of the research process. Ethical approval was obtained from the Regional Ethics Review Board, Umeå, Sweden (DNR 2017/79–31 M).

### 2.6 Rigour

To assure trustworthiness, each interview was individually coded, differences were discussed until consensus was reached. The conceptualization of subcategories and main categories were accurate documented. To establish credibility interpretations and conclusions were shared with the participants. To enhance confirmability each step in the procedure were systematically documented. The results may appear to be transferable to similar circumstances if the results are recontextualized to the current context (Polit & Beck, 2016).

## 3 RESULTS

The analysis process revealed two main categories (a) and five subcategories (b); (a) handling the situation, dealing with different opinions, (b) managing daily life, emotional roller coaster and general attitudes, own views and apprehensions, ignorance and lack of motivation. The main categories and subcategories are presented below, illustrated with quotations referenced to the participants.

### 3.1 Handling the situation

#### 3.1.1 Managing daily life

The participants expressed (from an early age) a stubbornness about managing their long‐term illness on their own, such as self‐administration of insulin, blood glucose tests, adjusting insulin and carb intake. Ten out of 12 participants said they were motivated, had knowledge of their own body reactions and a need to be in full control when planning daily arrangements. Eleven out of twelve participants reflected that they with age were prepared to take care of themselves and their illness. In general, they expressed they had full responsibility for their illness at early age. One participant: “It's always been there. I don't know anything else” (9).

Nine out of twelve participants experienced they could manage whatever they decided to; all that was required was a lot of scheduling. Planning was portrayed as something constantly present:

I wake up, check my blood glucose and eat if it's low, take insulin if it's high…. yeah yes then I munch anyway… but I`ll take more insulin if it's high and less if it's low and so… yes, I make myself ready for school and check my blood glucose after an hour or two. And that's what I do most of the time, like every half hour and then I count, no I don't count, I estimate carbs. I roughly know how much a potato is worth in carbs and then I insert insulin according to that. I always bring dextro‐energy in case of emergency. It drops very fast, so I always bring it and before I go to bed I always check and then eat if necessary (10).

The planning and structure were important both physically and mentally. All participants could picture their safety plans, for example, glucagon injections, sugar, dextro‐energy, blood glucose and ketone meter, etc. Nevertheless, four out of twelve did not fulfil the planning and structuring of the safety plans: “It's pretty simple to say I can do it! Of course, I can do better. But, it always turns bad again…cause it always turns better and some people got more focused and so do I… but it's so easy just to fall back and live like everybody else without diabetes” (5).

To keep control over their bodies nine out of 12 participants expressed they sat their blood glucose somewhat or way over normal in some situations: “It can be rather troublesome if I go low, so I think it´s better to lay high…to keep my mind clear” (11).

When speaking of healthcare professionals, nine out of twelve participants uttered satisfaction with the positive attitudes and welcoming from their nurses and physicians: “I thought I would get a gnarly bitch who just sat shouting at me. But then I got a marvellous nurse, really the best I've ever had… best that's ever happened to me” (1). Four out of twelve participants were confused about the structure of the different healthcare services. “Most important… in the child healthcare services, they tell you not to lay high… a must is below eleven, always and all the time. If you get over eleven, you must hurry to correct, correct and correct and then the new nurse… just like that! I think we will have to check out your lows before we correct your highs… I just love her…” (12).

One of the most exemplified tricky situations was consuming alcohol. One participant did not use alcohol, the other participants were afraid of losing control when using alcohol. One participant uttered learning the lesson by trial and error: “Because I know what it can cause, the alcohol itself. And what if I couldn't look after myself and put it in another person's hands? Hence, of course, it (got) more complicated when starting with alcohol and so on… Because ehh… today I have learned not a drop of insulin…the blood glucose drops like a stone” (5).

#### 3.1.2 Emotional roller coaster

In many cases, managing diabetes was expressed as stressful. Constant worries about the blood glucose, whether it was too high or too low: “It's like, earlier I checked my blood glucose far too seldom…they told me. Nowadays with this (*sensor*), I check it at least a hundred times per day and now the nurse told me I check it too often” (10). All participants shared a fear of losing control when having low blood glucose. Eight out of twelve managed that by constantly, or most of the time, placing themselves high in blood glucose: “No… I kind of get a depression every time I'm low so and that's kind of why I avoid it… It's like I immediately notice when I'm getting low and I think too much and that's really hard. Then suddenly it´s over and I think, What the !!!? Why did I get those thoughts?” (10).

One participant expressed there were no options, everybody expected that the responsibility lay on no one else, like it was obvious: “It's like… I did my thing, sort of… and maybe then I had a desire that my parents took their time and just asked… (3).”

Mood swings were stated as very common when having fluctuating blood glucose levels. Needing another person's assistance to correct low or high blood glucose was expressed as mentally strenuous: “First and foremost, I think I can suit myself! It's me and only me that put me in this situation and then I feel like a burden because I put me and others in this situation… I feel like a fool…” (1). One participant described frustration, anger and neglect. “Okay, I have bad blood glucose levels, then I just … it” (1).

### 3.2 Dealing with different opinions

#### 3.2.1 General attitudes and concerns

The participants explained that in most cases people were curious and interested in learning more about T1D: “I think it's pretty funny… people ask about my blood glucose levels, like… ohh eight, quite high.’ And I'm like, ‘Oh, no; I'm quite satisfied. I have just eaten my lunch: ‘I don't think people ask and have opinions of their own just to be rude. I think they ask because they have a genuine interest” (8).

Young adults with T1D reported several examples of being misunderstood or meet with an absence of knowledge. For example, one person ate a banana during a school lesson and was forced to leave the classroom because of eating in class: “I was hurt and disappointed by the way she did it. Also, maybe because she didn't understand that I had no choice. And the tone of her voice…” (2). A person who visited healthcare services was asked; “Are you allergic to something? No, I am not! I told her. At lunch time I asked the personnel if they had ordered any T1D food. But you told you had no allergies,” the personnel answered…” (8).

The participants exposed a great lack of general knowledge and understanding in people near them: “All my employers have asked if they should give me an injection if I get ill…” (4) or society in general. “Last month at the hospital, it's kind of very low, so I turned the pump off and pushed the alarm button. Then wait, wait and wait some more… suddenly a person shows up with a cup of tea! ‘Lemonade, sugar… give me something sweet!’ Oh my god. I nearly died at the hospital. Can you believe that?” (11).

#### 3.2.2 Own views and apprehensions

Eight participants out of twelve were not ashamed and did not feel forced to hide their illness: “My mum has always told me not to be ashamed of myself or my illness. According to her, I can do everything I want… and that's what always motivated me.” (7) However, four participants tried to hide their illness in some cases. It's like when you always get comments like: “Are you allergic to sugar? Are you sure this is the proper way for you to eat? What about lchf?” Oh my god; I decided not to tell people at my new job (8).

Half of the participants expressed they try to cover up the illness in certain situations: “…if it happens to happen at school. I really do not want to tell anyone. It's kind of embarrassing to tell people I'm low… cause sometimes I get too low and kind of tremble and… not able to walk or talk…just getting weaker and weaker” (3). The participants uttered the area of secondary school and in some cases high school as an area where they wished they were like everybody else, for example, healthy people. None of the participants could grab onto where this feeling came from, most likely from their own thoughts: “It's like… gah, I can do that later. It's no big deal not taking my injections right now. I can take care of that later on. I'd better run to catch up with my friends in the lunch cue…” (4).

The participants expressed great support from family and friends in the form of affirmation as well as mental and practical support: “I mean, he's driving me to the hospital and there is a silent agreement in how much he is supposed to ask and how much I share with him… and according to me, that's our winning concept” (8). Eleven out of twelve participants pictured their next of kin as helpful and understanding: “My boyfriend is fantastic. Sometimes at night if I act corny, he measures my blood glucose… just in case. He even keeps me company when meeting my diabetes team” (9). Despite this, in four cases, the participants described loneliness, anxiety and struggle: “It can kind of be a struggle to…sometimes I wish someone would have just asked…like, how is your diabetes today? A relative, a friend, just someone” (3).

A common trigger among the young adults was other people's unsubstantiated assumptions regarding the causes and treatment of Type1 diabetes: “It's so frustrating when people assume they know more than I do. (I) just, just can't stand it! That's something that triggers me” (9).

The participants illustrated a collective opinion that most people lack knowledge about T1D: “Look here…it's clear that people have no clue. It's sick…no knowledge. I wish people knew more. You see, if someone suffers from a stroke or epilepsy, it's like everybody knows how to act. But if you find a person with diabetes, no one knows. They think you are drunk” (12).

Ten out of twelve participants expressed satisfaction and delight with the way the nurses at the health services motivated and inspired them, talked to them as a human being and not just a disease: “…she also chitchats. She doesn't only focus on the illness… last time she even asked me about my summer… I like that” (5).

#### 3.2.3 Ignorance and lack of motivation

All participants expressed some sort of lack of motivation of the illness at some point in life: “That's when I panic. Oh no! I have to do something. I don't want to lose my legs. Then I keep it steady for one or two days, then I fall back again in mismanaging myself” (1). Nine participants described it lasted for a short period of time: “I remember from primary school…I found a drawing I made, with a text like “I hate diabetes” and a big red cross all over the picture” (12). Three participants expressed that the lack of motivation lasted several years and one participant that it still lasts. “I always bring my things, but I seldom take my insulin injections. It has always been like this, as long as I can remember” (11).

Six participants uttered they ceased insulin injections for special occasions: “And then I put my glucose level high… on purpose, to prevent low glucose levels and avoid the eating process. I wanted to avoid the injection process in public…” (7). For participants skipping insulin injections, it occurred (or occurs) in late teens or young adulthood: “But I thought it was very hard and then I just ignored it until I was able to go to the toilet or so… and sometimes I didn't take my injections for days” (5). Five participants out of the six skipping insulin injections expressed they changed their minds as they grew older: “Like… this is something that I never, ever will accept, to maybe I'll accept it someday, though I have no choice. The only way is to do the very best with the fucking situation” (4).

## 4 DISCUSSION

The results indicate that the participants were motivated had knowledge and in most cases were in control of planning and performing daily arrangements. This is in line with Olshansky et al. (2008) and Watts, O'Hara, and Trigg (2010), who indicate that some people seem to have the capacity to establish effective self‐ management behaviour. On the other hand, young adults undergo a great movement during these years. For example, leaving the parental home, graduating from high school and the transition from school‐ to work environments (Bryden et. al., 2001; Peters & Laffel, 2011). In line with Ozcan et al. (2014), our results show most participants found their own functional way of handling their long‐term illness where planning and structure was an essential part of their daily life. Nevertheless, half of the participants described they tried to cover the T1D up in certain situations, mostly related to other people's unsubstantiated assumptions. This type of challenges might partly explain the worsening of glycemic control during the years of 17–19 (Bryden et al., 2001; Insabella, Grey, Knafl, & Tamborlane, 2007).

The results revealed that participants were worried and sometimes scared of having low blood glucose. Related to low blood glucose, the participants were anxious about losing control of their bodies, the situation or even die, which also was shown in prior studies (Abdoli et al., 2017; Zoungas et al., 2010). The results pointed out the participants fear of being a burden to other people, for example, if they might end up in need of assistance. To avoid such situations, participants described maintaining their blood sugar higher than usual, some for special occasions and some for a longer period. Previous studies (Abdoli et al., 2017; Cryer, 2002; Martyn‐Nemeth et al., 2016) show that fear of hypoglycaemia is one of the major limiting factors in achieving optimal blood glucose control for persons living with T1D.

In line with prior research (Hillege, Beale, & McMaster, 2011; Kay, Krishnamurthy, Brodnicki, & Mannering, 2009; Peters & Laffel, 2011) the results revealed a range of distress and mental trigger causes, for example, thoughts of others discrediting, the anxiety of hypoglycemia, concerns regarding stigmatization or misunderstandings, day‐to‐day management, mood swings and future T1D related complications. Further, the results indicated that most people around young adults with T1D seem to be interested and curious to learn more about T1D. On the other hand, participants described people in general's lack of knowledge and understanding. As previously shown (Dougherty, 2015; Helgeson et al., 2014), lack of awareness and stigmatization from other people were common issues in daily life for persons with T1D. Studies (Bryden et al., 2003; Lin et al., 2010) indicated that young adult patients are especially vulnerable to psychological and psychiatric problems. Therefore, it is of great importance that young adults living with T1D are provided with continuous healthcare meetings, information, support and structured education (Balfe, Brugha, et al., 2013; MacDonald et al., 2013).

The period between 18 and 30 is characterized by exploration in various areas of life. The participants expressed support and assistance from near ones as much appreciated and helpful. Hanna (2012) and Helgeson et al. (2014) indicate that the management of T1D is primarily affected by support from friends and family. Also, Dougherty (2015), Helgeson et al. (2014), Sparud‐Lundin, Öhrn, & Danielson (2009) entitle that T1D itself implies a tremendous responsibility regarding self‐care management where parents, siblings, or significant others might be a great support throughout childhood. A few participants expressed lack of motivation and handling their daily T1D care. Hillard, Wu, Rausch, Dolan, & Hood, (2013) and Law (2013) imply this is a noteworthy cause of high blood glucose and that a pattern of chronic high blood glucose, in turn, leads to both short‐ and long‐term diabetes complications.

The findings indicate that most participants were satisfied with the support from healthcare services, that is, motivation and inspiration for self‐ care management. In line, the participants also illustrated that as they grew older, they gained a deeper understanding of their illness and self‐care management (Hirjaba et al., 2015; Sparud‐Lundin et al., 2009). Which in turn is in line with previous studies (Decoster, 2003; Peters & Laffel, 2011) suggesting that in young adulthood, the self‐care responsibility lies more with the individual contra childhood when the responsibility lay more on the parents.

Nevertheless, participants wished for continuous, more and deeper information and discussion about their illness, which is in line with previous studies (Nygren Zottermann, Skär, Olsson, & Söderberg, 2016; Polonsky & Fisher, 2015). Moreover, the results indicate the participants have shifting needs according to shifting life events (Balfe, Doyle, et al., 2013; Berg‐ Kelly, 2011; Weissberg‐Benchell, Wolpert, & Anderson, 2007).

### 4.1 Strengths and limitations

The patients’ narratives were the focal point at all times. Qualitative studies are suitable when seeking a deeper understanding of certain phenomenon. The sample size in this study was considered large enough to provide richness, as the interviews represent rich portrayals of the participants (Malterud et al., 2016). According to Sandelowski (1995), it is important that the sample is large enough to reach variation in the participants’ experiences.

The results of this study cannot be generalized but can be transferable to similar circumstances if the results are recontextualized to the current context (cf. Polit & Beck, 2016). One must keep in mind that the results from this study show results from a limited group of people living with T1D. Therefore, we do not claim to represent the opinions of all people with T1D. It is possible that people with interest in sharing their experiences agreed to participate and persons with entirely different opinions disagreed to participate, which might affect the results of this study. However, our results are in line with previous results, which in turn might indicate that our results may be transferable to some young adults living with T1D and possibly other patient groups with long‐term illness.

### 4.2 Implications for future research and clinical practice

Growing older, young adults with T1D gained a deeper understanding of their illness and wished for continuous, more in‐depth information and discussion about their illness. The findings contribute to the existing literature regarding young adults living with T1D. Further studies such as longitudinal qualitative studies to investigate the shape of T1D over time, that is, form a family of their own, work opportunities and state of long‐term illness. Quantitative studies would be of interest to test the quality of life and wellbeing in young adults with T1D.

The young adults with T1D are a unique patient group due to their everyday lives, with wide‐ranging commitments to their health. Therefore, we need to further look into the work for proper guidelines for patients and healthcare providers. Healthcare providers’ contra patient's responsibility for information, communication and collaboration. Finally, the ethical considerations regarding the inflexible transition from child to adult healthcare services. The results raise the thought of taking a family perspective when caring for people living with T1D. Decisions made in adult care services affect the entire family. Family meetings might be a useful tool to ensure cooperation and integration of family perspectives for the benefit of all persons involved.

## 5 CONCLUSION

The results revealed various findings about motivation, knowledge and control of long‐term illness. However, the participants expressed they were anxious about losing control of their bodies, the situation or even die. They also revealed a fear of being a burden to other people, that is, friends, family or co‐workers. The results showed each participants found their own functional way of handling and living with their long‐term illness, planning and structure were an essential part of their daily life. Mental trigger causes such as other peoples stigmatization, misunderstandings, own mood swings and future T1D‐related complications were also revealed in the results section. These findings might be useful as guidance to nurses and other practitioners in their daily work, as well as increased knowledge in friends, family, relatives and significant others.

## CONFLICT OF INTEREST

No conflict of interest has been declared by the authors.

## AUTHOR CONTRIBUTIONS

Study design: ÅC, SS, data collection: ÅC, SS, analysis: ÅC, SS and manuscript preparation: ÅC, SS.
